# The dynamic relationship between multiple team membership and individual job performance in knowledge‐intensive work

**DOI:** 10.1002/job.2260

**Published:** 2018-01-16

**Authors:** Hendrik J. van de Brake, Frank Walter, Floor A. Rink, Peter J. M. D. Essens, Gerben S. van der Vegt

**Affiliations:** ^1^ Faculty of Economics and Business University of Groningen Groningen the Netherlands; ^2^ Faculty of Economics and Business Studies Justus‐Liebig‐University Giessen Giessen Germany

**Keywords:** dynamic relationships, job performance, latent change score modeling, multiple team membership, within‐person relationships

## Abstract

Many employees in modern, knowledge‐based organizations are concurrently involved in more than one team at the same time. This study investigated whether a within‐person change in such individual multiple team membership (MTM) may precede and may be predicted by changes in an employee's overall job performance. We examined this reciprocal relationship using longitudinal archival data from a large knowledge‐intensive organization, comprising 1,875 employees and spanning 5 consecutive years. A latent change score model demonstrated that an increase in an employee's MTM was associated with a subsequent decrease in his or her overall job performance evaluations. By contrast, an increase in job performance was associated with a subsequent increase in an employee's MTM. Moreover, our results indicated that although an increase in an individual employee's MTM initially decreases his or her job performance, in the long run, this increase in MTM was associated with higher job performance. Together, these results suggest a dynamic association between an individual employee's MTM and his or her overall job performance, such that these variables are mutually connected in a highly complex manner over time.

## 1 INTRODUCTION

In an attempt to use scarce human resources as effectively and efficiently as possible, knowledge‐based organizations increasingly rely on flexible project teams in which memberships are frequently shared, shifted, and dissolved (Mortensen, 2014). Within such contexts, many individuals work on more than one project at the same time (O'Leary, Mortensen, & Woolley, 2011), enabling various teams to concurrently benefit from their expertise. For example, individual research and development employees often work simultaneously on several project teams, with each team utilizing their specific knowledge and contributions (Bertolotti, Mattarelli, Vignoli, & Macrì, 2015). Similarly, many academics are concurrently involved in multiple research and teaching teams. Scholars have estimated that such multiple team membership (MTM) occurs among at least 65% of employees across a wide range of occupations (Mortensen, Woolley, & O'Leary, 2007; O'Leary, Mortensen, et al., 2011).

As a result of this development, there is growing scholarly interest in the consequences of MTM (Wageman, Gardner, & Mortensen, 2012). Much of this research has focused on the team level of analysis, illustrating for example that members' simultaneous involvement in various other teams may shape a focal team's performance outcomes (e.g., Bertolotti et al., 2015; Cummings & Haas, 2012). Importantly, however, multi‐teaming may also distinctly influence individual employees' work experiences and behaviors (Mortensen et al., 2007). Compared with more traditional contexts with clearly defined and delimited team memberships, individuals may face unique opportunities and challenges from their involvement in multiple organizational teams (i.e., individual MTM; O'Leary, Mortensen, et al., 2011). Beyond team‐level performance implications, it therefore seems critical to understand how an individual employee's MTM can shape his or her job performance (i.e., an employee's overall contributions toward the organization's goal achievement across tasks and teams; Borman & Motowidlo, 1997).

The scarce empirical research on individual MTM has generally focused on MTM's relatively proximal, psychological, and cognitive consequences (e.g., employees' project overload and work engagement; Pluut, Flestea, & Curşeu, 2014; Zika‐Viktorsson, Sundström, & Engwall, 2006). These studies have created important insights, and they make it plausible to assume that an individual's MTM may also shape his or her job performance as a more distal—yet vitally important—outcome variable. Importantly, however, the existing research has not directly examined MTM's role for an employee's overall job performance. What is more, theoretical arguments about MTM's potential performance consequences have remained ambiguous. Some scholars have suggested that MTM can provide employees with important resources that may enhance their job performance, for example, by increasing their social network or creating unique learning opportunities (Hansen, 1999; Vedres & Stark, 2010). By contrast, other researchers have argued that MTM imposes considerable demands upon employees that may lower their job performance, for example, by forcing employees to regularly relocate and/or to shift between distinct tools, tasks, and technologies (O'Leary, Mortensen, et al., 2011; Zika‐Viktorsson et al., 2006). As a result, the performance implications of MTM remain unclear.

Beyond ambiguity about the possible performance benefits and detriments of MTM for individual employees, the current literature cannot answer key questions about the direction of MTM‐performance linkages. Research on team staffing (Hackman & Wageman, 2004) and individuals' preferred work characteristics (LePine, Podsakoff, & LePine, 2005) suggests that there is a distinct possibility of reciprocal causation, such that the relationship between individual MTM and an employee's overall job performance may also flow in the opposite direction. Changes in an employee's job performance might shape his or her subsequent MTM, in particular, because (a) managers tend to select high performers when staffing their teams and (b) performance growth may increase an employee's confidence and motivation to join additional team settings. Consequently, employees whose performance has improved may experience a subsequent increase in their MTM. The link between an individual's MTM and job performance may thus be more intricate than previously believed, with these constructs either amplifying or counteracting each other over time (cf. Maruyama, 1963; Weick, 1979).

The present research uses a novel, dynamic approach to address the above issues. We draw from the notion that increasing an employee's MTM may both augment and diminish job‐related resources and build on two resource‐based theories (i.e., social capital and conservation of resources theory; Hobfoll, 1988; Lin, 1999) to develop competing hypotheses about the way changes in individual MTM may relate with subsequent changes in employees' overall job performance. We pit these competing perspectives against each other, using data from 1,875 knowledge workers. Whereas prior work has typically used cross‐sectional, between‐person designs to examine the association between MTM and performance‐related outcomes (e.g., Chan, 2014; Cummings & Haas, 2012; Pluut et al., 2014), we adopt a longitudinal, within‐person study design to investigate this linkage over time. This approach enables us to examine whether changes in an employee's overall job performance may relate to subsequent changes in his or her MTM (O'Leary, Mortensen, et al., 2011; O'Leary, Woolley, & Mortensen, 2011) and allows us to investigate potentially reciprocal relationships between these variables over time.

Taken together, the present investigation strives to realize several contributions to the MTM literature. Extending previous theory and research on team‐level MTM and on the psychological consequences of individual‐level MTM, we aim to increase our understanding of how individual employees' engagement and disengagement with multiple concurrent teams relates to their overall job performance over time. More specifically, our goal is to resolve existing ambiguities about the linkage between individuals' MTM and job performance by clarifying (a) whether MTM's performance benefits or drawbacks will prevail, (b) how this relationship unfolds over time, and (c) whether an employee's MTM may serve both as an antecedent and as a consequence of his or her job performance. To achieve this goal, we introduce a longitudinal, within‐person perspective to the study of individual MTM that investigates the relationship between changes in employees' MTM and job performance over time. This dynamic perspective moves beyond the static approaches prevalent in most of the MTM research to date, promoting theory advancement by enabling unique insights into the complex, potentially reciprocal within‐person relationships between individual employees' MTM and job performance.

## 2 THEORY AND HYPOTHESES

### 2.1 Individual employees' MTM

Prior studies have typically conceptualized MTM at the team level of analysis, such that MTM represents the extent to which a focal team's members are, on average, involved in other teams as well (e.g., Bertolotti et al., 2015; Mortensen, 2014). Importantly, we hold that such team‐level MTM's origins are located at the individual level of analysis, denoting the extent to which individual employees simultaneously are members of more than one (project) team (O'Leary, Mortensen, et al., 2011). Empirically, this is reflected in the number of teams to which an individual allocates working time during a specific period (e.g., on a weekly basis; O'Leary, Mortensen, et al., 2011). Recent studies have found that it is rather common for individuals in some occupations, especially in knowledge‐based work, to simultaneously be a member of up to eight or nine teams (Cummings & Haas, 2012; Pluut et al., 2014). Moreover, theorists have emphasized the potential relevance of such individual MTM, arguing that it may create unique experiences, demands, and possibilities at work that decisively shape an employee's job‐related attitudes, behaviors, and outcomes (e.g., Mortensen et al., 2007; O'Leary, Mortensen, et al., 2011). As such, this study examines MTM at the individual level of analysis, defining the concept as an individual employee's number of concurrent team memberships.

An employee's individual MTM differs from related concepts such as multitasking (Leroy, 2009) and task switching (Monsell, 2003). Multitasking, for instance, refers to a situation in which an employee simultaneously carries out two or more tasks, whereas MTM reflects the number of concurrent teams to which an individual allocates time and attention (Salvucci & Taatgen, 2008). This distinction is important for two reasons. First, MTM does not necessarily involve frequent task switching (and vice versa). Even an employee with high MTM may avoid excessive changes between different tasks, for example, by compartmentalizing his or her working time into predictable sequences (e.g., working for the first team on Monday and Tuesday and the second team on Wednesday and Thursday; Monsell, 2003). Second, both multitasking and task switching usually refer to how employees deal with multiple individual task assignments (Salvucci & Taatgen, 2008). In contrast, MTM is inherently social and interactive, as relevant assignments are carried out interdependently within multiple team contexts (Van Der Vegt, Van De Vliert, & Oosterhof, 2003; Wageman et al., 2012). As such, individual MTM is a unique phenomenon, with causes and consequences that cannot be directly derived from existing knowledge on multitasking and task switching.

### 2.2 Individual MTM as an antecedent of job performance: A resource‐based perspective

As noted before, changes in an employee's MTM may go along with unique advantages and disadvantages that, ultimately, can enhance or diminish his or her overall job performance (Mortensen et al., 2007; O'Leary, Mortensen, et al., 2011). In particular, we propose that an increase in MTM may either improve access to, or distract from, key resources required for an employee's effective performance at work. Hence, we draw from two prominent theoretical perspectives that both highlight an employee's job‐related resources (i.e., valued entities that serve to achieve job‐related ends; Hobfoll, 1989) as key determinants of individual performance. Social capital theory (Kwon & Adler, 2014), on the one hand, points toward possible resource gains that can be achieved through complex interpersonal work arrangements, such as MTM (Lin, 1999). Conservation of resources theory (Hobfoll, 1988, 1989), on the other hand, emphasizes possible resource losses that can arise from changes in an employee's working conditions (for an overview, see Halbesleben, 2006). Accordingly, the first perspective suggests that an increase in MTMs may increase an employee's overall performance levels, whereas the latter perspective suggests that an increase in MTM may decrease an employee's job performance. As these conceptual approaches lead to competing hypotheses about the role of MTM changes for subsequent performance developments, they therefore allow us to conceptually disentangle both the positive and negative aspects of an employee's concurrent memberships in multiple teams.

#### 2.2.1 A social capital perspective on MTM's consequences

Social capital theory suggests that an employee's social network (i.e., his or her interpersonal connections with coworkers; Borgatti & Foster, 2003) entails valuable interpersonal resources (e.g., knowledge, information, instrumental, and social support) and it defines social capital as an employee's capacity to access and utilize these resources (Lin, 1999, 2002). Such social capital is known to be a key factor that can facilitate an employee's job performance, because individuals with greater social capital can more easily draw on the resources required to promote their performance outcomes (Kwon & Adler, 2014; Thompson, 2005). Within innovative, nonroutine work contexts, it is particularly useful to establish new connections across distinct organizational subunits (e.g., teams), as these linkages provide access to a greater diversity of perspectives and information, political connections across various parts of the organization, and differing types of expertise (Cross & Cummings, 2004; Lin, 1999).

Based on this notion, it seems plausible to argue that an increase in an employee's MTM enables additional productive connections across different teams, thus promoting the social capital needed to achieve higher performance levels. Indeed, by its very definition, MTM requires individuals to cooperate with other employees from multiple distinct teams, often with diverse areas of expertise (O'Leary, Woolley, et al., 2011). Hence, increasing MTM may enable employees to work with a greater number of previously unfamiliar colleagues, project leaders, and clients, thus providing access to valuable resources that are embedded within different teams and offering the unique opportunity to transfer these resources across team contexts (Choi & Thompson, 2005; Tasselli, Kilduff, & Menges, 2015; Vedres & Stark, 2010). An increase in MTM may, for example, expose an employee to new knowledge sources that spark his or her creativity (Grant, 1996; Perry‐Smith, 2006), familiarize the employee with innovative work practices that could be useful in other team settings as well (e.g., by sharing best practices; Burt, 1992), and create opportunities to establish new and meaningful relationships with coworkers in various parts of the organization (Hansen, 1999; Van der Doef & Maes, 1999). Individuals with stable MTM levels over time, by contrast, have to rely on their existing social resources to a greater extent and, thus, may find it more difficult to realize such opportunities for creativity, learning, and knowledge exchanges.

Taken together, this reasoning suggests that MTM may represent a distinct source of social capital (beyond an employee's sheer number of interpersonal connections; Borgatti & Foster, 2003). As such, increasing MTM may provide unique performance advantages for the respective individuals.

Hypothesis 1a An increase in an employee's MTM is related to a subsequent increase in his or her overall job performance.

#### 2.2.2 A conservation of resources perspective on MTM's consequences

Importantly, however, there are also good conceptual reasons to expect a fundamentally different pattern. Conservation of resources theory, in particular, argues that people seek to obtain, retain, and protect valuable resources that help them to perform effectively, and that stress occurs when such resources are threatened or depleted (Brotheridge & Lee, 2002; Hobfoll, 1988). In organizational settings, the most widely studied of these resources relate to employees' perceived ability to control important aspects of their work (Skinner, 1996; Van der Doef & Maes, 1999) and to the time and attention employees are able to direct toward completing their tasks (Hobfoll, 1989; Thompson, 2005). Empirical research has demonstrated that substantial losses of these resources can diminish an employee's overall functioning (e.g., by invoking stress and decreasing task efficiency; Halbesleben, 2006; LePine et al., 2005; Rich, Lepine, & Crawford, 2010). As outlined below, we argue that increasing MTM may directly affect an employee's perceived control over his or her tasks across various teams and, relatedly, the time he or she has available to meet each team's demands. It therefore appears plausible, from this perspective, to suggest that an increase in MTM may decrease an employee's subsequent job performance.

First, increasing MTM may reduce an employee's ability to control important aspects of the job. An increase in MTM implies that an individual's tasks and interdependencies are spread out over a greater number of concurrent teams (Mortensen, 2014; Wageman et al., 2012), such that he or she encounters a greater variety of task requirements and interpersonal expectations from additional colleagues, managers, and clients across diverse team settings (O'Leary, Mortensen, et al., 2011). Accordingly, increases in MTM require an employee to adjust to new team roles and adapt to the unique characteristics of each respective team (Cummings & Haas, 2012; Mortensen et al., 2007). This may obstruct an employee's ability to effectively comprehend the novel procedures, knowledge domains, and social demands relevant for each team's task accomplishment, potentially lowering the employee's sense of control and, consequently, reducing his or her overall job performance (Hobfoll, 1989; 2002; Kauppila, 2014; O'Leary, Woolley, et al., 2011).

Second, an increase in MTM decreases the amount of time an employee can spend on a team before having to move on to the next assignment, in a different team context (Mortensen et al., 2007; Rich et al., 2010). This may pose considerable challenges for the effective organization of an employee's task routines and time scheduling. Each additional team membership, for example, increases the amount of effort required to catch up with work done in an employee's absence, and it decreases his or her available time to adjust to distinct tools, tasks, and technologies used within each specific team (O'Leary, Mortensen, et al., 2011; Zika‐Viktorsson et al., 2006).

Together, this reasoning suggests that an increase in MTM may deplete an employee's performance potentials (Halbesleben, 2006; Hobfoll, 1989). This rationale is consistent with scholarly arguments pointing to MTM's demanding and highly complicated nature as a key source of job strain, lowered satisfaction, and reduced work engagement (Kauppila, 2014; Leroy, 2009; Pluut et al., 2014). Consequently, our second hypothesis is

Hypothesis 1b An increase in an employee's MTM is related to a subsequent decrease in his or her overall job performance.

### 2.3 Individual MTM as a consequence of job performance

So far, we have discussed changes in MTM as an antecedent of an individual employee's job performance. Although this reasoning appears theoretically plausible, it seems equally possible that the MTM‐performance linkage follows a reversed direction. Specifically, an increase in an employee's performance may associate with an increase in his or her subsequent MTM because increased performance may result in a greater number of requests to join additional teams and increase an employee's willingness to accept such requests. 1In contrast to MTM's performance consequences, we see little theoretical rationale to expect both positive and negative linkages between an employee's overall job performance and subsequent MTM. Hence, we focus on the positive association between performance increases and subsequent MTM changes in the following, and we do not develop competing hypotheses for this association.


Research on employee staffing and team member selection suggests that an employee's prior job performance may shape his or her attractiveness as a prospective team member (Hinds, Carley, Krackhardt, & Wholey, 2000). When looking for qualified individuals to staff a specific (project) team, it is clear that team leaders and project managers typically strive to attract employees with high potential (Kerzner, 2013)—and individual employees' prior performance trajectories offer an important indication of this potential. Individuals who have exhibited marked performance improvements in the past, in particular, are likely to be motivated and willing to exert effort, and they have demonstrated the ability to learn and adapt (Borman & Motowidlo, 1997). Such individuals implicitly signal, therefore, that they have the potential to develop themselves and, hence, to handle additional task demands and projects (Cummings & Haas, 2012). Similar effects may occur in self‐managing teams, where members themselves take responsibility for staffing decisions (Alper, Tjosvold, & Law, 1998; Chuboda, Wynn, Lu, & Watson‐Manheim, 2005). In these teams, the existing members typically look for new teammates through informal social connections and previous work experiences (Casciaro & Lobo, 2008). In doing so, a candidate's prior performance improvements and associated reputation gains may again play a critical role (D'Souza & Colarelli, 2010; LePine & Van Dyne, 2001).

Another reason why a reversed direction in the MTM‐performance linkage is possible is that employees who have experienced improved overall job performance in the past may be more inclined to proactively seek and accept memberships in additional teams. In this regard, research suggests that positive performance feedback increases an employee's confidence in his or her ability to manage complex and demanding working conditions (Jex, Bliese, Buzzell, & Primeau, 2001; Kim & Hamner, 1976). Consequently, such employees may be more open to new challenges that create opportunities for future growth, as compared with employees whose job performance has stagnated or even decreased (LePine et al., 2005; O'Leary, Mortensen, et al., 2011). Additional team memberships may provide them with such challenges (e.g., through social network expansion, learning opportunities, and increased task diversity; Bertolotti et al., 2015; O'Leary, Mortensen, et al., 2011). Indeed, a qualitative study found that MTM “provides employees with opportunities to shape their careers by joining projects related to expertise they have or want to develop” (Mortensen et al., 2007: 5).

Taken together, this reasoning suggests that employees who have recently increased their performance will receive and accept a disproportionally higher number of invitations for concurrent team memberships. Consequently, we propose

Hypothesis 2 An increase in an employee's overall job performance is related to a subsequent increase in his or her MTM.

### 2.4 The dynamic relationship between changes in MTM and overall job performance

Whereas our competing Hypotheses 1a and 1b propose that an increase in an individual employee's MTM will either positively or negatively associate with his or her subsequent job performance, Hypothesis 2 predicts that an employee's increasing job performance will positively associate with an increase in his or her subsequent MTM. Taken together, these hypotheses point toward potentially dynamic, reciprocal relationships between changes in an individual's MTM and overall job performance. Corroborating Hypotheses 1a and 2, on the one hand, would suggest that increases in MTM and job performance reinforce each other in a positive, “deviation‐amplifying” feedback loop that spirals both variables toward higher levels over time (Weick, 1979, p. 73). A decrease in individual MTM or job performance, by contrast, would then pose a major risk factor that could trigger a downward‐spiraling relationship.

Corroborating Hypotheses 1b and 2, on the other hand, would suggest that changes in individual MTM and job performance are dynamically related in a “deviation‐counteracting” feedback loop (Weick, 1979, p. 74), such that increases in one variable would instigate decreases in the other, inducing relative stability (despite minor oscillations) in the long run. This would imply that increasing MTM neutralizes an employee's previous performance improvements (and vice versa), thus leading toward stagnating MTM and performance levels.

Clearly, these divergent patterns of reciprocal relationships would carry important implications for our theoretical understanding of the linkage between individual MTM and job performance over time. Hence, we will closely scrutinize these potential dynamics in the following. Given that our competing predictions in Hypotheses 1a and 1b leave considerable ambiguity about the expected shape of these associations, however, we decided to not develop formal hypotheses in this regard.

## 3 METHODS

### 3.1 Sample and data collection

To test our hypotheses, we used a sample of knowledge workers from an organization of applied research with roughly 3,500 employees, located in the Netherlands. Work within this organization was structured along (contract) research projects, with project managers attracting funding and subsequently staffing temporary teams with suitable employees. In addition, employees had the opportunity to proactively apply for specific team memberships by approaching the respective project managers (who retained final say over staffing decisions). Although the organization did not publicly communicate individuals' formal performance appraisals, project managers were generally well aware of relevant employees' performance reputation. In part, this was because work within the organization was highly collaborative and required extensive exchange of information and materials with employees across multiple teams, departments, and knowledge domains. As such, MTM was a relatively common phenomenon within our host organization, offering an ideal setting to examine the linkage between individual employees' MTM and overall job performance.

We obtained longitudinal data from the organization's personnel records, spanning five consecutive years (2008–2012). Specifically, the organization provided weekly work hour registrations for all 3,348 individuals permanently employed with the organization. These employees were obliged to register the number of work hours spent for different project teams in a very detailed manner. Among other things, the organization used this information for billing purposes and to calculate project costs; hence, project managers closely monitored the accuracy of these registrations. Further, the department of human resources supplied us with demographic information and yearly performance evaluations for all employees. Given our study's focus, we excluded individuals who, due to the nature of their tasks, were not involved in specific project teams (i.e., lower level administrative personnel and general managers). Finally, an employee's inclusion in this study required the availability of complete demographic information as well as data on both MTM and performance for at least one time point each (Li, Fay, Frese, Harms, & Gao, 2014).

Our final sample comprised 1,875 employees that carried out applied research in project teams. These employees were well educated (i.e., they had at least a bachelor's degree) and predominantly male (74%); their mean age was 41 years (*SD* = 10.5), and they had been working with the organization for an average of 11 years (*SD* = 9.6) at the beginning of our study period. Moreover, most of the employees in our sample worked on a full‐time basis (81%); they worked on approximately 13 projects per year, with an average of 2.7 projects per week (range = 1–10). Almost all of the sample employees (96%) were, at some point during the 5‐year study period, members of more than one project team at the same time.

As is common in longitudinal research, there were missing data across the different time points. Of the 1,875 sample employees, complete data were available for 1,218 individuals in Year 1; 1,337 individuals in Year 2; 1,452 individuals in Year 3; 1,463 individuals in Year 4; and 1,497 individuals in Year 5. For 947 employees, complete data were available across all study years. The missing data in the present sample predominantly resulted from individuals that, during our study period (a) entered or left the organization, (b) moved to a position within the organization that did not involve work in research projects (e.g., departmental leadership), or (c) were absent for an extended period of time (e.g., due to sickness, pregnancy, or a sabbatical). Following recommendations of Graham (2009) and Nakai and Ke (2011), we used maximum likelihood estimation for models with partial missing data when testing the study hypotheses through latent change score models (as outlined below), enabling us to fully utilize all information available in the present sample. We note that the results and conclusions remained virtually unchanged, however, when using a listwise deletion procedure for hypotheses testing. 2Scholars have noted that missing data in longitudinal studies can cause biased parameter estimates if it arises from systematic participant attrition (e.g., due to inferior performance evaluations; Graham, 2009). Importantly, however, dismissal of low‐performing employees is unlikely to represent a substantial source of attrition in the present sample. The participants in our sample were employed under permanent (i.e., nontemporary) contracts which, under Dutch labor law, are relatively difficult and costly to terminate and, thus, provide high job security. Hence, lower performance ratings would typically result in improvement interventions and reduced salary increases, rather than layoffs.


### 3.2 Measures

We captured employees' MTM and job performance for each of the 5 years during our study period. Identical procedures were used each year to measure these constructs.

#### 3.2.1 MTM

Similar to prior research (e.g., Chan, 2014; Pluut et al., 2014), we measured an employee's MTM as the number of concurrent project teams in which he or she was actively involved. Contrary to this earlier work, however, we used archival data (rather than survey measures) to obtain a detailed indication of an individual employee's number of active team memberships. Specifically, the employees in our sample reported their team‐related working time on a weekly basis through the formal work hour registrations mentioned above, and we used these archival data to capture the number of teams to which an individual allocated working time during a specific week. 3Because we were interested in individuals' memberships within multiple teams, work hours for projects with less than three members (less than 1% of all projects) were excluded (Dyer, 1984). To match the annual job performance measure available within personnel records (see below), we subsequently used this information to calculate an employee's average MTM within each year of the study period. Our measure therefore represents an individual employee's annual average number of teams per week. Conceptually, this implies that MTM increases when an individual becomes actively involved (i.e., spends time) in a greater number of teams (cf. Pluut et al., 2014). Mirroring recent reports of increasing MTM across many organizations and occupations (Mortensen et al., 2007)—and corroborating the relevance of our dynamic approach toward examining MTM—our data illustrate a slight trend toward increased multi‐teaming during the study period (see Table 1).

**Table 1:** Means, standard deviations, and Pearson correlation coefficients

Variable	Mean	*SD*	1	2	3	4	5	6	7	8	9	10	11	12	13
1. Organizational tenure (years)	11.13	9.61													
2. Gender (F = 0, M = 1)	.74	.44	.22**												
3. Salary (standardized)	.00	1.00	.49**	.24**											
4. FTE	.94	.11	.02	.24**	–.13**										
5. MTM (Y1)	2.68	1.15	.13**	−.02	.10**	.05									
6. MTM (Y2)	2.74	1.20	.07**	−.02	.06*	.07*	.75**								
7. MTM (Y3)	2.77	1.20	.04	−.03	.07**	.07**	.63**	.74**							
8. MTM (Y4)	2.73	1.85	.04	–.06**	.05*	.07**	.55**	.60**	.73**						
9. MTM (Y5)	2.84	1.23	.05*	–.06*	.04	.04	.52**	.58**	.64**	.78**					
10. Performance (Y1)	3.30	.58	–.25**	–.06*	–.15**	.10**	.08*	.08**	.10**	.08*	.08**				
11. Performance (Y2)	3.28	.60	–.24**	–.05*	–.17**	.11**	−.01	.08**	.06*	.06*	.02	.44**			
12. Performance (Y3)	3.31	.65	–.25**	−.03	–.18**	.17**	.02	.05	.08**	.12**	.10**	.35**	.53**		
13. Performance (Y4)	3.37	.61	–.28**	−.02	–.24**	.10**	.04	.07*	.07*	.09**	.08**	.32**	.36**	.45**	
14. Performance (Y5)	3.30	.58	–.24**	−.04	–.24**	.14**	−.01	.07*	.06*	.06*	.10**	.26**	.24**	.29**	.43**

*Note*. *N* total = 1,875 employees. FTE = full‐time equivalents; MTM = multiple team membership.

* *p* < .05.

** *p* < .01.

#### 3.2.2 Overall job performance

At the end of each year, the host organization's human resource management system required departmental supervisors to assess each of their direct reports' overall job performance. These supervisors were responsible for 5–25 employees within their departments (mean = 14), and they typically met with these employees and relevant project leaders on a daily to biweekly basis. As such, supervisors had a relatively detailed and accurate view of their individual employees' overall performance.

Following prior research (e.g., Bommer, Johnson, Rich, Podsakoff, & MacKenzie, 1995; Cross & Cummings, 2004), we used supervisors' formal appraisal scores to operationalize individual employees' yearly overall job performance. Beyond their own assessment of an employee's technical proficiency, planning and organizational skills, and research output, supervisors were asked to incorporate into their evaluations (a) feedback provided by project leaders about the quality of an employee's performance outcomes and (b) annual peer assessments by direct colleagues and/or customer assessments (if available). Supervisors used a standardized evaluation form to rate each individual employee's overall job performance on a 5‐point scale, with 1 representing the *worst* possible evaluation (i.e., substantial need for improvement) and 5 indicating the *best* possible evaluation (i.e., highly effective and well‐functioning). The organization used these formal performance appraisal scores, in part, to determine employees' salary increases and promotions. As such, appraisal outcomes had direct practical relevance for the employees in our sample.

#### 3.2.3 Control variables

We considered a number of covariates that may relate to individual employees' MTM and/or overall job performance. Previous studies have suggested, in particular, that supervisory performance evaluations may be biased on the basis of employees' *gender* (Inesi & Cable, 2015), *organizational tenure* (Ng & Feldman, 2010), and *salary* (Cleveland, Murphy, & Williams, 1989). For example, supervisors may expect greater contributions toward organizational goals from employees with longer work experience and higher pay (Sturman, 2003). Hence, even if supervisors have relatively accurate information about an individual employee's actual job performance (as was the case in this study context; see above), this information might translate into different performance ratings, depending on an employee's organizational tenure or salary. Moreover, organizational tenure may shape an employee's MTM, because individuals with higher tenure may develop specific skills that are useful for a greater number of teams (Cummings & Haas, 2012). We therefore included these variables as potential controls. Because the host organization was opposed to publishing detailed salary information, this particular variable was available in *z*‐standardized form only. Finally, we anticipated that full‐time employees had more time available for work in additional teams, as compared with part‐time employees. We therefore incorporated an individual's weekly working time (in full‐time equivalents [FTE]) as an additional covariate (Pendleton, 2010). 4To further explore this potential biasing factor, we repeated our hypotheses tests using more restricted samples that only included full‐time employees. The results and conclusions from these supplementary analyses remained virtually unchanged. To preserve statistical power, we therefore report the results based on the full sample in the following. Scores on the control variables were very stable over time (or changed by a fixed amount each year). To reduce the complexity of our models, we therefore created time‐invariant control variables by taking individual means across all available time points (Acemoglu, Johnson, Robinson, & Thaicharoen, 2003).

### 3.3 Data analysis

We employed latent change score (LCS) modeling (using Mplus version 7.11; Muthén & Muthén, 1998) to test our hypotheses (McArdle, 2009). Researchers have used this method to examine the potentially reciprocal nature of the relationships between, for example, work characteristics and changes in personality (Li et al., 2014), cognitive training exercises and improvements in critical reasoning (McArdle & Prindle, 2008), and life events and behavioral problems (Malone et al., 2004). In this study, we employed Grimm, An, McArdle, Zonderman, and Resnick's (2012) extension of the LCS framework to examine how within‐person changes in one variable relate to subsequent changes in a second variable. This allowed us to examine whether an increase in an employee's MTM was related with a subsequent increase (Hypothesis 1a) or decrease (Hypothesis 1b) in job performance and, simultaneously, whether an increase in an employee's job performance was related with a subsequent increase in his or her MTM (Hypothesis 2). In other words, we examined within‐person changes in MTM as both an antecedent and a consequence of within‐person changes in an individual employee's overall job performance (Hackman & Wageman, 2004; Hinds et al., 2000), thus testing the dynamic, potentially reciprocal relationship between these variables.

**Figure 1:**
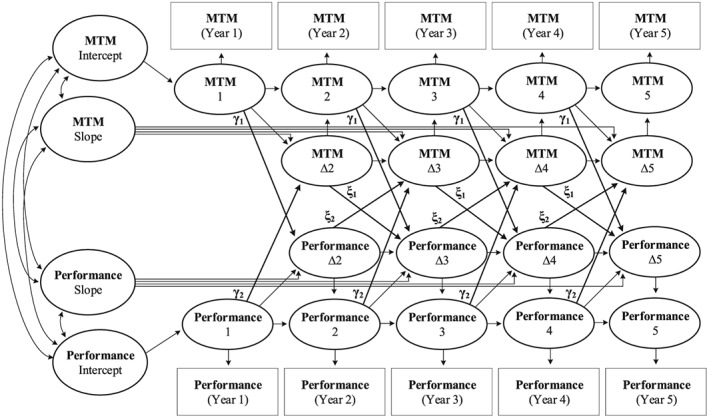
A latent change score model for MTM and overall job performance. Note: Adapted from McArdle (2009) and Grimm et al. (2012). Control variables are not shown. MTM = multiple team membership

The LCS model used to test our hypotheses is visualized in Figure 1 (see Grimm et al., 2012, for a detailed description of each component of the model, as well as the Mplus scripts used to fit the model to the data). A key feature of an LCS model is that it uses a structural equation modeling framework to model change as a latent variable, representing an increase or decrease in the observed scores for each variable between two adjacent time points. These within‐person changes (e.g., ∆ job performance, T2‐T3) are predicted, then, by changes in a second variable at an earlier time point (e.g., ∆ MTM, T1‐T2). In addition, the model controls for changes that occur due to an employee's level of MTM or performance (e.g., MTM, T1). Together, this allowed us to examine whether within‐person changes in MTM were indeed related to within‐person changes in an employee's overall job performance and vice versa. As is common when using LCS models, all estimates were assumed to be equal across time points (Grimm et al., 2012; McArdle, 2009). Moreover, we controlled for the relationships of gender, organizational tenure, salary, and FTE, on the one hand, with individual differences in MTM and job performance, on the other, when estimating our model. 5None of the controls predicted within‐person changes in MTM and job performance, and adding these relationships substantially decreased the overall fit of our model (cf. Hu & Bentler, 1999). Hence, we only included the controls to account for individual differences in MTM and job performance.


## 4 RESULTS

### 4.1 Descriptive statistics

Table 1 reports descriptive statistics and bivariate correlations for all variables across all five time points. As shown, an employee's MTM and overall job performance were positively correlated within each year of our study period (*r* range *=* .08 to .10; all *p* < .01). Moreover, an employee's performance was consistently positively correlated with MTM in the subsequent year (*r* range *=* .06 to .12; all *p* < .05), whereas MTM associated with subsequent performance in only two out of the 5 years (*r* range *= −*.01 to .07). Note, however, that these bivariate correlations reflect between‐person associations. An adequate test of our within‐person hypotheses, in contrast, requires longitudinal techniques of data analysis, as presented below.

Regarding the temporal stability of the variables in our sample, we note that the correlations between MTM across two adjacent years varied between .73 and .78 (all *p* < .01), suggesting moderate‐to‐high MTM stability over time (which is relatively common in longitudinal studies; see Usami, Hayes, & McArdle, 2016). Similarly, job performance exhibited moderate stability, with correlations across subsequent years ranging from .43 to .53 (all *p* < .01). These correlations indicate that the study variables were relatively stable at the between‐person level, suggesting that there was little variation between the sample employees in the MTM and performance shifts they experienced. Nevertheless, it is possible that individual employees experienced significant within‐person changes in their MTM and performance during the study period (Grimm et al., 2012). As such, the present stability levels do not prevent further examination of within‐person changes in MTM and performance over time (Li et al., 2014).

Finally, regarding potential covariates, gender was significantly related to both MTM and job performance at two time points (*r* range *=* −.05 to −.06; all *p* < .05). Moreover, employees' organizational tenure, salary, and FTE were negatively related to performance across all time points (*r* tenure range *=* −.24 to −.28; *r* salary range *=* −.15 to −.25; *r* FTE range *=* .10 to −.17; all *p* < .01), suggesting that supervisors' performance ratings were positively biased toward less experienced employees with lower salaries. Although counterintuitive at first glance, this finding is consistent with prior research that has argued supervisors to hold heightened expectations toward more experienced employees and, thus, to more critically assess their job performance (Cleveland et al., 1989; Sturman, 2003).

### 4.2 Hypotheses tests

Table 2 presents the results of an LCS model that tested the relationships between changes in MTM and subsequent changes in overall job performance (ξ_1_), and between changes in performance and MTM changes (ξ_2_). This model (which includes both between‐ and within‐person associations) provided a good fit to the data (χ^2^ = 368.00, *df =* 68, *p* < .01; RMSEA = .05, CFI = .95, SRMR = .06; cf. Hu & Bentler, 1999), and it fit the data significantly better than a basic LCS model that only included between‐person relationships (χ^2^_difference_ = 143.45, *df*_difference_ = 4, *p* < .01).

**Table 2:** Parameter estimates for a latent change score model with multiple team membership and job performance

Parameter	Estimate (*SE*)
Intercepts and slopes	
Mean intercept MTM	2.83 (.05)**
Mean slope MTM	5.67 (3.35)
Mean intercept performance	3.20 (.03)**
Mean slope performance	−.77 (1.04)
Correlations	
Slope MTM with intercept MTM	1.47 (.41)**
Slope performance with intercept performance	−.01 (.03)
Slope MTM with intercept performance	.15 (.11)
Slope performance with intercept MTM	−.34 (.15)*
Slope MTM with slope performance	−.52(.37)
Intercept MTM with intercept performance	.04 (.02)*
Controls	
Organizational tenure ➔ MTM level	.02 (.00)**
Gender ➔ MTM level	−.02 (.04)
Salary ➔ MTM level	.08 (.02)**
FTE ➔ MTM level	−.60 (.06)**
Organizational tenure ➔ performance level	−.01 (.00)**
Gender ➔ performance level	.02 (.02)
Salary ➔ performance level	−.04 (.01)**
FTE ➔ performance level	.26 (.04)**
Level MTM ➔ change in performance (γ_1_)	.33 (.15)*
Level performance ➔ change in MTM (γ_2_)	−.58 (.73)
Hypotheses tests	
Change in MTM ➔ subsequent change in performance (ξ_1_)	−.71 (.31)*
Change in performance ➔ subsequent change in MTM (ξ_2_)	9.95 (1.92)**

*Note*. *N* total = 1,875 employees. FTE = full‐time equivalents; MTM = multiple team membership.

* *p* < .05.

** *p* < .01.

Hypothesis 1a predicted that an increase in an individual's MTM would positively relate with a subsequent change in his or her overall job performance, whereas Hypothesis 1b predicted an MTM increase to negatively associate with a subsequent performance change. The results presented in Table 2 refute Hypothesis 1a but support Hypothesis 1b, illustrating that an increase in MTM related to a subsequent decrease in job performance (ξ_1_ = −.71, *p* < .05). Hypothesis 2 predicted that an increase in job performance would associate with a subsequent increase in MTM. As shown in Table 2, the respective relationship is indeed positive and significant (ξ_2_ = 9.95, *p* < .01), thereby supporting Hypothesis 2. Together, these results suggest that changes in an individual employee's MTM and his or her overall job performance dynamically relate to each other in a negative, deviation‐counteracting feedback loop (cf. Weick, 1979). That is, a within‐person increase in job performance relates to a subsequent increase in MTM, but an increase in MTM relates to a subsequent decrease in job performance.

### 4.3 Additional findings

Beyond the reciprocal relationship between an individual's changes in MTM and job performance, our LCS model also assessed (a) whether an employee's MTM level was associated with subsequent performance changes and (b) whether an employee's performance level was associated with subsequent MTM changes. As shown in Table 2, an employee's overall job performance level (i.e., individual performance differences between persons) was not significantly related with changes in his or her MTM (γ_2_ = −.58, *p* > .10). An employee's MTM level, by contrast, was significantly and positively related to subsequent changes in his or her job performance (γ_1_ = .33, *p* < .05). Hence, employees who worked in more teams at the same time experienced greater increases in performance than employees working in less teams at the same time. Although we did not explicitly formulate hypotheses for such “level‐to‐change” relationships, these additional findings have important implications for the overall pattern of linkages between individual MTM and job performance.

**Figure 2:**
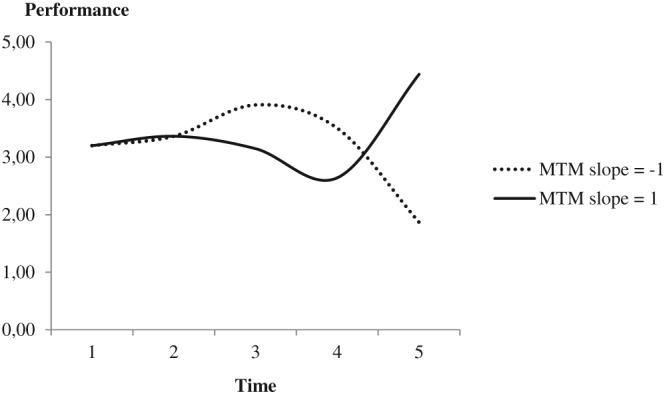
Predicted performance trajectories for employees with constant increases or decreases in MTM over time. MTM = multiple team membership

To illustrate the complex interplay of the various parameters in our overall LCS model, Figure 2 depicts two exemplary performance trajectories, namely, for an employee that experienced a constant increase in MTM over time (MTM slope = 1; solid line) and for an employee that experienced a constant decrease in MTM (MTM slope = −1; dashed line). With the exception of the respective MTM slope differences, all other parameters used to generate the predicted trajectories were identical, as reported in Table 2 (cf. Grimm et al., 2012). As shown, both of these exemplary performance trajectories therefore start at the same level but, subsequently, develop differential patterns over time. Assuming a constant rate of increasing MTM (i.e., MTM increases with 1 at each time point, in addition to the change predicted by other parameters in the model), the solid line indicates that, after a brief period of relative stability, a marked performance decrease ensued. This reflects the detrimental performance effects of a within‐person increase in MTM predicted in Hypothesis 1b. Over time, however, constant increases in MTM logically result in a relatively high overall MTM level and, as illustrated by the sharp performance increase between Years 4 and 5, the positive performance effects associated with an individual's MTM level eventually prevailed over the negative effects associated with MTM changes. Assuming a constant rate of decreasing MTM, by contrast, the dashed line in Figure 2 indicates an initial performance increase, because the observed negative association between changes in MTM and an individual's job performance implies that a decrease in MTM improves individual performance. Eventually, however, the relatively low MTM level resulting from continuously decreasing MTM rendered these performance gains untenable, as evidenced by the sharp drop in performance after Year 3.

## 5 DISCUSSION

Many of today's employees are simultaneously involved in more than one team (Mortensen et al., 2007; O'Leary, Mortensen, et al., 2011). In the present manuscript, we examined the dynamic relationship between such MTM and individual employees' overall job performance, drawing on a 5‐year longitudinal sample of knowledge workers. From a within‐person perspective, we found that changes in an employee's MTM were negatively associated with subsequent performance changes, whereas changes in an employee's job performance were positively related with subsequent changes in his or her MTM. It appears, therefore, that individual MTM and job performance changes are mutually linked with each other in a deviation‐counteracting feedback loop (Maruyama, 1963; Sterman, 2000; Weick, 1979). Moreover, from a between‐person perspective, we found that an employee's overall MTM level was positively related with job performance changes, such that employees with a greater number of concurrent team memberships may enjoy performance advantages. Hence, it is clear that the relationship between individual MTM and job performance is highly complex, and an adequate understanding of this linkage requires full consideration of its underlying dynamics. The following sections highlight the implications of such a dynamic perspective, as reflected in the present findings, for both theory and organizational practice.

### 5.1 Theoretical implications

The present findings make several important contributions to the literature on teamwork, in general, and MTM, in particular. The overwhelming majority of the empirical research on organizational work teams has focused on stable teams with clear boundaries, with individuals conceptualized as members of one particular team (Wageman et al., 2012). In reality, however, many teams in modern organizations represent unstable, dynamic entities with blurry boundaries (Mortensen, 2014), and individuals often work in multiple teams at the same time (O'Leary, Mortensen, et al., 2011). Accordingly, this study addresses repeated calls for research “that analyzes team member time allocation in a more dynamic way, examining how members engage and disengage with particular teams over time” (Cummings & Haas, 2012, p. 338; see also Mathieu, Tannenbaum, Donsbach, & Alliger, 2014; Wageman et al., 2012). Moving beyond prior team‐level performance considerations (e.g., Bertolotti et al., 2015; Mortensen, 2014), our research illustrates the distinct, reciprocal relationships between an individual employee's concurrent memberships within different teams, on the one hand, and his or her overall job performance, on the other.

Our findings also help to resolve a puzzle presented by prior theorizing on the consequences of MTM for individual job performance. Indeed, conservation of resources theory (COR) and social capital theory suggest that there are both potential performance benefits (O'Leary, Mortensen, et al., 2011) and detriments (Pluut et al., 2014) associated with MTM. Rather than contradicting each other, our results suggest that COR and social capital theory speak to different aspects of the dynamic linkage between individual MTM and job performance. Consistent with COR theory, the negative association between changes in MTM and subsequent performance changes may reflect the initial depletion of psychological resources (cf. Hobfoll, 1988). The positive association between an individual's MTM level and performance changes, however, may reflect longer‐term benefits. Indeed, our results suggest that initial resource investments (albeit costly) may eventually strengthen an employee's resource base and promote his or her functioning at work (the second principle of COR theory; Brotheridge & Lee, 2002; Chen, Westman, & Hobfoll, 2015). These longer‐term effects are in line with social capital theory, which suggests that employees may benefit from the interpersonal resources embedded within their social networks once they have developed adequate connections with colleagues across various teams (Kwon & Adler, 2014). The present research contributes to the literature by integrating these perspectives using a dynamic perspective on relationship between MTM and individual performance.

Importantly, these complex results highlight a real risk for incorrect conclusions about the MTM‐performance association that may arise when neglecting the underlying dynamics. It appears that MTM, by itself, is neither harmful nor helpful for an employee's performance. Rather, the costs and benefits of MTM hinge on the time frame under consideration. Whereas the process of increasing an employee's MTM may initially harm his or her job performance, high MTM levels may eventually enable substantive performance improvements. It seems vital, therefore, to consider individual MTM from a longitudinal perspective that combines within‐person and between‐person approaches, considering both the extent to which an employee's MTM changes over time and an employee's overall level of MTM.

Moreover, our 5‐year longitudinal data allowed us to examine the directionality of the MTM‐performance linkage, thus reconciling previous theoretical claims that have alternatively cast MTM as an antecedent (e.g., Chan, 2014) or as a consequence (e.g., Cummings & Haas, 2012) of an employee's job performance. Beyond the complex MTM‐to‐performance linkages discussed before, our within‐person results show that increasing performance may subsequently promote an individuals' MTM as well. So, paradoxically, increasing performance may carry within itself the potential of both future performance decrements and future performance benefits in MTM settings.

### 5.2 Limitations and future research directions

Although our research has several methodological strengths (e.g., 5‐year longitudinal data from a relatively large sample of employees; an MTM measure based on objective data), we acknowledge some limitations that should be considered when interpreting the results. Our sample consisted of employees from a single organization and cultural context, for example, and it therefore seems worthwhile to examine our findings' generalizability to other organizations, industries, and countries. Furthermore, we used supervisory assessments to capture individual employees' job performance, based on a scale developed by the host organization, because objective performance information was not available (cf. Bommer et al., 1995; Cross & Cummings, 2004). Although these supervisory assessments had high practical relevance (e.g., providing important inputs for decisions about an employee's remuneration and career progress), future research might examine MTM's relation with alternative and/or more specific (e.g., objective, customer‐rated, and team‐member rated) performance measures.

Relatedly, our research focused on an employee's overall job performance, neglecting the employee's differences in performance across teams. It may be worthwhile to control for these performance differences in future research. Moreover, future research might extend the insights from our study by developing a finer‐grained model that explicates the linkage between MTM and specific performance dimensions. It is possible, for example, that MTM's performance consequences differ, depending on the job performance dimensions under consideration (e.g., efficiency vs. creativity), or that different performance dimensions differ in how strongly they impact an employee's MTM.

Furthermore, despite our longitudinal design, causality claims should be regarded with caution, in particular because we were not able to study individuals' MTM and job performance from the very beginning of their employment. To more confidently conclude that prior performance changes cause changes in an individual's MTM (or that an employee's MTM causes performance changes), it would be helpful to more comprehensively track the formation of an individual's membership in multiple teams. Certain employees, for example, may be specifically hired (e.g., based on certain skills or personality characteristics; LePine & Van Dyne, 2001) or trained (e.g., through socialization programs; Chao, O'Leary‐Kelly, Wolf, Klein, & Gardner, 1994) to work in more than one team at the same time. Hence, their work may be characterized by high MTM levels from the outset, rather than by gradual, performance‐induced increases in MTM. Furthermore, we used relatively long, 1‐year intervals to measure both MTM and job performance. As such, we may have primarily captured broad, longer‐term associations between these variables. For a more detailed understanding of the underlying dynamics, future research may benefit from examining MTM‐performance linkages using shorter time intervals.

Future research could also examine potential mediating variables and boundary conditions in the linkage between MTM and individual employees' performance. Building on COR and social capital theory (Hobfoll, 1988; Lin, 1999), we argued that specific social and psychological resources may explicate MTM's performance advantages and disadvantages, yet this study context did not allow us to directly capture these mechanisms. The literature could therefore benefit from a further examination of such mediating factors, for example, by directly investigating the potential resource losses (e.g., lower perceived control; Skinner, 1996) and social capital advantages (e.g., increased network centrality; Borgatti & Foster, 2003) associated with increasing MTM.

Similarly, we did not examine potential boundary conditions for the present relationships (beyond the time frame under consideration), and this may be a worthwhile subject for future research as well. Specific features of the work situation, such as support from colleagues and/or supervisors (Cohen & Hoberman, 1983) or personality traits such as proactivity (Li et al., 2014) may enable employees to address the challenges and utilize the opportunities associated with increasing MTM. Relatedly, it may be useful to study the mediating mechanisms that explain how employees respond to increased MTM. Research on employees' coping with stressful work events points to personal growth and resource accumulation as potentially relevant mediator variables (e.g., Cohen & Hoberman, 1983; Hobfoll, 1988; Updegraff & Taylor, 2000). Investigating such moderating and mediating factors may add more context‐ and person‐specific richness to the longitudinal dynamics examined in our study.

Finally, MTM's performance consequences may hinge not only on the number of an employee's concurrent team memberships (i.e., MTM quantity, as captured in this study) but also on qualitative differences between teams (i.e., MTM variety; O'Leary, Mortensen, et al., 2011; O'Leary, Woolley, et al., 2011). Whereas some employees may be concurrent members in similar teams, others may be immersed in a greater variety of team contexts (e.g., representing diverse organizational areas). The latter employees are exposed to larger variations in social contexts and task procedures, potentially aggravating MTM's performance disadvantages by promoting role conflicts and work overload (Kauppila, 2014; Rizzo, House, & Lirtzman, 1970). On the other hand, greater MTM variety might also promote the performance potentials derived from an employee's concurrent team memberships, enabling access to a greater variety of knowledge and resources (Vedres & Stark, 2010). Moreover, better performing employees may not only become members of a greater number of teams but might also self‐select into more challenging and varied team contexts (Hackman & Wageman, 2004) or might be assigned to more diverse teams (because these employees may be more attractive for teams across diverse departments; Cummings, 2004). Future empirical research that moves beyond the present focus on quantitative MTM differences could include such variations in MTM quality to further promote theory development in this relatively nascent line of inquiry.

### 5.3 Practical implications and conclusion

The complex, dynamic relationship between individual employees' MTM and overall job performance uncovered in our study has important practical implications. Based on our findings, managing employees' MTM to achieve optimal performance appears as a challenging task that requires considerable resource investments from both individual employees and the organization. As illustrated in Figure 2, increasing an employee's MTM is likely to initially trigger substantial performance detriments. Consequently, employees and organizations may be tempted to shy away from such changes, retaining or even reducing an employee's MTM to yield short‐term performance benefits. Figure 2 also shows, however, that increasing MTM may, in the long run, induce pronounced performance gains that may outweigh initial downsides. Hence, it seems worthwhile to consider increasing MTM as a resource investment that, eventually, result in important performance advantages. Both employees and their organization may benefit from accepting the costs of developing an employee to increasingly work in MTM settings, enabling them to reap the longer‐term advantages higher MTM levels entail.

For individual employees, this implies that they should view novel team memberships as resource investments and maintain their involvement in new, additional teams, even if this may initially harm their personal resources and performance potential. Moreover, from an organizational perspective, it implies that managers should not hold immediate performance losses resulting from increasing MTM against an employee, refraining from organizational reprimands and/or premature reduction of the employee's MTM. By persisting through the difficulties that increasing MTM entails and by actively supporting employees during this initial phase (e.g., through coaching or mentoring; Cohen & Hoberman, 1983), organizations may eventually achieve a more versatile workforce that can draw from relevant work experiences, insights, and social connections across multiple teams to attain superior performance levels.

Altogether, our study contributes to an integrative understanding of an employee's overall performance as both an antecedent and a consequence of his or her MTM, and it underlines the importance of a dynamic, longitudinal approach in examining these linkages. We hope these findings will stimulate further research on the temporal dynamics of contemporary team memberships, and individual‐level MTM in particular, thus helping organizations to effectively manage such complex teamwork arrangements.
